# A multi‐informant and multi‐polygenic approach to understanding predictors of peer victimisation in childhood and adolescence

**DOI:** 10.1002/jcv2.12063

**Published:** 2022-02-23

**Authors:** Jessica M. Armitage, Geneviève Morneau‐Vaillancourt, Jean‐Baptiste Pingault, Till F. M. Andlauer, Stéphane Paquin, Stéphanie Langevin, Mara Brendgen, Ginette Dionne, Jean Séguin, Guy Rouleau, Frank Vitaro, Isabelle Ouellet‐Morin, Michel Boivin

**Affiliations:** ^1^ School of Psychological Science University of Bristol Bristol UK; ^2^ School of Psychology Université Laval Québec City Québec Canada; ^3^ Division of Psychology and Language Sciences University College London London UK; ^4^ Department of Neurology Klinikum rechts der Isar School of Medicine University of Munich Munich Germany; ^5^ Department of Psychology The Pennsylvania State University State College Pennsylvania USA; ^6^ Department of Criminology Université de Montréal Montreal Québec Canada; ^7^ Department of Psychology Université du Québec à Montréal Montreal Québec Canada; ^8^ Department of Psychiatry and Addictology University of Montreal Montreal Québec Canada; ^9^ Department of Neuorlogy and Neurosurgery McGill University Montreal Québec Canada; ^10^ School of Psycho‐Education Université de Montréal Montreal Québec Canada

**Keywords:** individual vulnerabilities, multi‐informant approach, peer victimisation, polygenic scores

## Abstract

**Introduction:**

Peer victimisation is a prevalent occurrence in childhood and adolescence and can often have long‐lasting consequences. Previous research using polygenic scores (PGSs) have revealed various genetic vulnerabilities as predictive of victimisation in childhood. However, findings were based on self‐report and may therefore be influenced by varying self‐perceptions. Previous investigations also focused on average victimisation across childhood, and thus do not capture variability in polygenic predictability over time. The present study, therefore, aimed to investigate associations between PGSs and victimisation using separate and combined reports from teachers and peers in childhood, as well as self‐reports in later adolescence to explore trajectories of victimisation.

**Methods:**

Data were derived from the Quebec Newborn Twin Study. Participants were assessed for victimisation using self‐reports from 7 to 17 years and using teacher ratings and peer nominations between 7 and 10 years (*n* = 536). Ten PGSs related to mental health, cognitive abilities and physical traits were examined as possible predictors of victimisation using linear regressions and growth curve models.

**Results:**

Findings revealed that PGSs associated with victimisation are consistent across informants, but to varying extent according to estimated effect sizes. Self‐reported victimisation was predicted by PGSs related to mental health, while PGSs related to cognitive and physical traits had larger effect estimates when predicting teacher‐ and peer‐reported victimisation. The PGS for educational attainment was consistently negatively associated with victimisation across informants, producing the largest effect estimates (*β* = −.104, 95% CI = −.169 to −.039) when predicting a multi‐informant measure of victimisation. No PGS predicted changes in victimisation over time.

**Conclusion:**

While the PGS for educational attainment is a robust predictor of victimisation, many PGSs are differentially associated with victimisation depending on the informant. Such findings highlight the need to pay close attention to the phenotypic assessment of victimisation, and show that using multiple informants can both strengthen and provide unique insight into how associations may occur.

## Graphical abstract

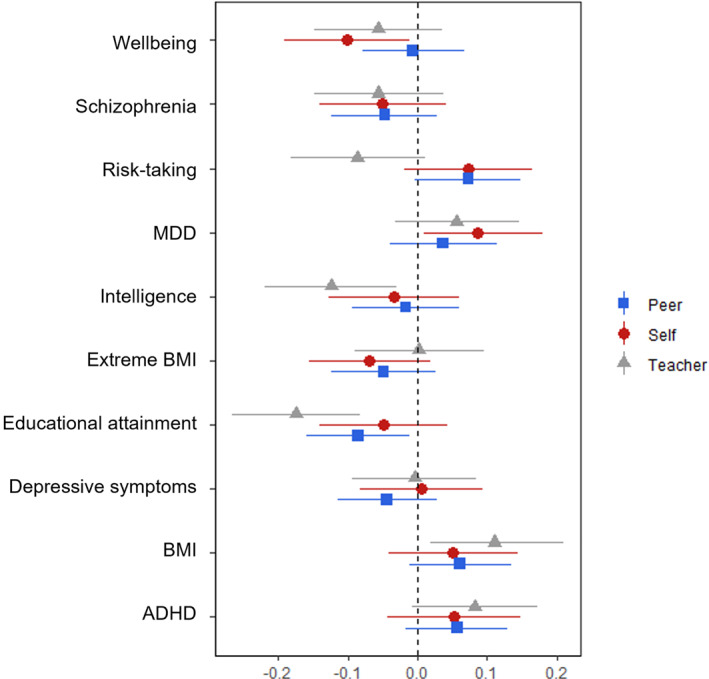

A host of genetic predispositions have been shown to predict the likelihood that someone becomes a victim of bullying. We show for the first time, that these vulnerabilities and their association with peer victimisation vary depending on the informant. Such findings show that using multiple informants can help strengthen predictions of developmental outcomes based on genetic information, as well as provide unique insight into paths by which these may occur.

**Key points**

- Previous research using polygenic scores (PGSs) has shown that peer victimisation is predicted by various traits and vulnerabilities. The extent to which these genetic dispositions reflect self‐perception biases, however, is unknown.
- This study shows for the first time that genetic predictors of peer victimisation are influenced by the informant, with self‐reports more associated with genetic risk for mental health problems, and teacher‐ and peer‐reports more closely linked to cognitive and physical traits. PGSs associated with educational attainment, however, is a robust predictor of victimisation across informants.
- Our findings highlight the need to pay close attention to the phenotypic assessment of victimisation and demonstrate that using multiple informants can strengthen predictions of developmental outcomes based on PGSs.

## INTRODUCTION

Peer victimisation occurs when an individual is repeatedly exposed to physical, verbal or relational aggression from the same peer or groups of peers (Olweus & Limber, 2010). Over 15% of adolescents are subjected to frequent experiences of victimisation, with often negative repercussions for physical and mental health (Armitage et al., 2021; Copeland et al., 2013). These adverse effects, however, have been shown to lessen over time (Singham et al., 2017), highlighting the potential for resilience. To understand and prevent victimisation leading to negative outcomes, it is helpful to ascertain the developmental processes underlying victimisation. Identifying such vulnerabilities early on could inform prevention strategies by targeting those most at risk.

A host of individual vulnerabilities have been shown to correlate, sometimes predictively, with the likelihood of peer victimisation. These include pre‐existing mental health problems (Cook et al., 2010), lower intelligence (Verlinden et al., 2014), and being socially withdrawn (Boivin et al., 2010; Morneau‐Vaillancourt et al., 2021). Such findings, however, typically derive from observational phenotypic studies and are thus limited in their ability to identify predisposing factors that may drive predictive associations. This is because such designs are subject to reverse causation, making it difficult to infer if the identified factors casually impact subsequent risk. One approach to tackling reverse causation and triangulating results in observational research is to use genetically informed methods.

The genetic sequence is fixed from conception (baring mutations), which implies that associations between genetic factors and exposures are free from reverse causation. The twin design, which disentangles the relative contributions of genetic and environmental factors, can thus be used to elucidate underlying mechanisms and tackle with more confidence the direction of associations (Kretschmer et al., 2018). Findings have revealed that the risk of being victimised is substantially accounted for by genetic factors, with heritability estimates around 77% (Johansson et al., 2020). A significant part of this genetic liability appears to be shared with other behavioural risks for victimisation, including disruptive behaviours (Boivin et al., 2013a), conduct disorder (Musci et al., 2018) and psychotic symptoms (Pergola et al., 2019). This means individuals at a higher genetic risk for these problems are also more likely to be victimised by their peers. Such knowledge provides important insight into the genetic and environmental processes underlying peer victimisation and later difficulties.

Although twin research provides a general, latent assessment of the genetic aetiology of developmental outcomes, such designs are blind to specific genetic variants. To measure genetic sources of variance more directly, researchers can use polygenic scores (PGSs). PGSs are derived from largely populated genome‐wide association studies (GWASs) whereby millions of genetic variants are scanned to identify those associated with a phenotype. Such common variants are summed and incorporated into an individual‐specific propensity score, each weighted by the magnitude of their association with the targeted phenotype (Dudbridge, 2013). The resulting PGS can be used to investigate associations with the original phenotype, or with other traits, with often just moderate sample sizes needed to attain sufficient power (Dudbridge, 2013). Multiple PGSs can also be considered in unison to evaluate their independent and unique effects.

Since no large‐scale GWAS has been published on peer victimisation, we still know little about the molecular genetic aetiology of victimisation. Using PGSs is therefore a relevant tool to explore this. Investigating multiple PGSs is particularly important when studying experiences like victimisation, for which there are known multi‐factorial risk factors. One study adopted this multi‐polygenic approach to assess the contribution of 35 PGSs to the risk of peer victimisation (Schoeler et al., 2019). The PGSs were associated with mental health, cognition, personality, and physical features, and both their individual and joint contributions to victimisation were examined. In total, 10 PGSs were significantly associated with victimisation in univariate analyses, among which 5 were independently predictive in a multivariable model. These were PGSs related to depression, ADHD, risk‐taking, BMI, and intelligence, with all but intelligence positively associated with victimisation. Although limited in magnitude due to the nascent nature of PGSs, the pattern of associations was consistent with previously identified risk factors for peer victimisation.

An important shortcoming of the Schoeler et al. (2019) study, however, is that it relied on self‐reported victimisation. Self‐reports are the most common method for determining victimisation in schools (Vivolo‐Kantor et al., 2014), but concerns have been raised regarding the sole reliance on this assessment source (Boivin, Brendgen et al., 2013b). Children who experience peer difficulties often vary in their perceptions of depression, with rejected children more likely to report stronger feelings of depression (Boivin et al., 1994). This is likely to be heightened among individuals at a higher genetic risk to depression, meaning individuals at an increased genetic risk may be more likely to report negative peer treatment. To better ascertain the putative effects of relying on self‐reports when investigating predictors of peer victimisation, it is necessary to consider reports beyond the victim.

As peers witness a broad range of social interactions within the school environment (Craig & Pepler, 1997), and thus provide crucial insight into peer relationship difficulties. Such reports are also commonly derived from multiple respondents, meaning responses based on just a single item have high reliability (Hodges et al., 1997). To complement peer‐reports, teacher evaluations can also be used. Teachers play an important role in the management of classroom bullying (Yoon & Bauman, 2014) and are less influenced than peers by relational biases (Ladd & Kochenderfer‐Ladd, 2002). However, teachers may lack access to relevant incidents in peer interactions, particularly as children get older. Considering the various perspectives of the child, his or her peers and teachers is therefore key to validly assess childhood victimisation occurrence.

Another key limitation of Schoeler et al. (2019) study is the use of an aggregate score of victimisation across ages 8–13 years. The transition from childhood to adolescence is however characterised by significant physical, psychological, and social changes. Accordingly, individuals may vary in their level of exposure to victimisation across this transition, and the characteristics that put a person at risk may change over that time. This means that a genetic vulnerability for a given trait may play out differently in adolescence than in childhood. No study, however, has examined the role of genetic vulnerabilities in victimisation across development.

One way to investigate changes in polygenic predictiveness is to use a repeated measures approach. Growth‐curve modelling involves extracting longitudinal data to study how individual trajectories unfold over time in comparison to a population. Such an approach is based on multiple time points and therefore reduces potential measurement error. This could mean that even with smaller samples, analyses outperform those based on just one occasion. The aim of the current study was thus to use the power of the polygenic and longitudinal design to overcome previous limitations and document more comprehensively the role of genetic vulnerabilities in predicting peer victimisation.

Drawing on the work of Schoeler et al. (2019), our first aim was to replicate and extend their findings by documenting whether teacher‐ and peer‐reports can provide a unique insight into predictors of victimisation. To do this, we draw comparisons between different informant reports, as well as create an overall multi‐informant measure of victimisation. A second aim was to expand the developmental coverage to document possible variations with age. This was achieved using self‐reports of victimisation beyond childhood to include later adolescence.

## METHODS

### Sample

Phenotype and genotype data were from the prospective longitudinal Quebec Newborn Twin Study (QNTS). Families with twins born between 1995 and 1998 in the Greater Montreal area were contacted, and 662 (67%) agreed to participate when the twins were 5 months (Boivin et al., 2019). Twins have since been followed annually, with individual, social, family, and school characteristics collected. Full details are reported in Boivin et al. (2019). Parental and child informed consent was obtained from the ethics review board at Université Laval, Quebec.

The present study is based on assessments at ages 7, 10, 12, 13, 15 and 17 years. At 7 and 10 years, we use data from twins with relevant self‐, peer‐, and teacher‐reports, and from those at age 12 with self‐ and teacher‐reports. From secondary school onwards, assessments were based on self‐report. These were deemed more accurate because students have multiple classrooms and teachers for different subjects. Further information is provided in Table 1, and information about attrition can be found in Table S1.

**TABLE 1:** Victimisation scores based on age and informant

	*N*	%Male	*M*(SD)	Min	Max
Age 7					
Self‐reported	527	51.0	0.71(0.52)	0.00	2.00
Teacher‐reported	476	51.3	0.26(0.37)	0.00	2.00
Peer‐reported	465	50.8	−0.05(0.96)	−2.19	2.90
Overall victimisation^a^	536	51.3	−0.03(0.73)	−1.39	2.33
Age 10					
Self‐reported	480	51.3	0.68(0.42)	0.00	2.00
Teacher‐reported	436	50.7	0.25(0.38)	0.00	2.00
Peer‐reported	418	50.7	0.04(0.95)	−1.35	3.83
Overall victimisation^a^	536	51.3	−0.01(0.74)	−1.33	3.14
Age 12					
Self‐reported	459	49.2	0.48(0.34)	0.00	2.00
Teacher‐reported	375	46.1	0.21(0.38)	0.00	2.00
Age 13					
Self‐reported	450	48.9	0.37(0.33)	0.00	1.89
Age 15					
Self‐reported	417	48.0	0.21(0.25)	0.00	1.67
Age 17					
Self‐reported	429	47.6	0.18(0.23)	0.00	1.25
Mean scores across ages					
Self‐reported childhood^b^	507	49.3	0.60(0.32)	0.00	1.94
Teacher‐reported childhood^c^	448	50.1	0.24(0.29)	0.00	1.50
Peer‐reported childhood^d^	518	51.2	−0.04(0.81)	−1.73	3.72
Self‐reported adolescence^e^	450	47.3	0.25(0.21)	0.00	1.15

*Note*: Peer‐reported have been *z*‐standardised.

^a^ Overall victimisation represents factor analysis scores based on peer‐, teacher and self‐reports.

^b^ Self‐reported childhood composite based on assessments from 7, 10 and 13 years.

^c^ Teacher composite based on assessments from 7, 10 and 12 years.

^d^ Peer composite based on assessments from 7 to 10 years.

^e^ Self‐reported adolescent composite based on assessments from 13, 15 and 17 years.

## MEASURES

### Self‐reported victimisation

Self‐reported victimisation was assessed using structured interviews. At each time point, participants answered five questions from the previously validated Self‐report Victimisation Scale (Ladd et al., 2002). Items were based on both direct and indirect experiences (see Table S2). Responses were recorded on a three‐point scale (0 = Never, 1 = Sometimes, 2 = Often) and averaged at each time point. The scales had adequate internal consistency (Cronbach's alpha (*α*); 7: *α* = .67; 10: *α* = .72; 13: *α* = .67; 15: *α* = .84; and 17 years: *α* = .83). Correlations between self‐reports across time ranged from low to moderate, with higher correlations observed between those assessed in closer proximity (see Table S3).

To assess self‐reported victimisation across either childhood or adolescence, we created an overall mean victimisation score for both periods. For childhood, this was computed using at least two scores from ages 7, 10 and 12 years. For adolescence, the mean was based on two or more time points from ages 13, 15 and 17 years. Correlations between the mean childhood composite and the adolescent composite were *r* *=* 0.40.

### Teacher‐reported victimisation

Teacher reports were recorded using responses to the following statements: ‘In the past 6 months, how often would you say that the child was (1) made fun of by other children, (2) hit or pushed by other children, and (3) called names by other children’. Responses were all recorded on a three‐point scale (0 = Never, 1 = Sometimes, 2 = Often) and averaged. Such items showed adequate internal consistency (7: *α* = .70; 10: *α* = .82; 12: *α* = .71). As per the self‐reports, a mean score was computed using at least two teacher rated scores at ages 7, 10 and 12 years. Correlations between teacher‐reports across time were slightly higher than those found using self‐reports (Table S3).

### Peer‐reported victimisation

Peer‐reported victimisation was assessed using peer nominations at ages 7 and 10 years. Photographs of all children in a class were handed out to participating children, who were asked to circle photos of two classmates ‘…who get called names most often by other children’, and ‘…who are often pushed and hit by other children’. These statements were adapted from the victimization subscale of the previously validated modified Peer Nomination Inventory (Perry et al., 1988). The total number of nominations received from all classmates for each item was calculated for each participant. Scores for the two statements were highly correlated at both time points (7: *r* = 0.50, 10: *r* = 0.65), and averaged at each age. According to standard procedures for peer nomination data (Cillessen & Rose, 2005), items were *z* standardized within classroom to account for differences in classroom size. A mean score was created by averaging the mean scores from ages 7 and 10 years, which correlated at *r* = 0.29.

### Overall victimisation

A two‐factor confirmatory analysis (CFA) was used to combine scores from the different informants (self, peers, and teachers) to provide an indication of overall victimisation. This was carried out using data for all three informants, at ages 7 and 10 years (see Figure S1 in the Supporting Information). The first overall victimisation factor at age 7 years was composed of reports from the self, peers, and teachers. Similarly, the second factor was composed of all three informants' reports at age 10 years. Both factors were allowed to correlate. The analysis was conducted on the full QNTS sample to maximise power (and not just on the genotyped subsample; *N* = 1049). To account for dependency between twins, the analysis was conducted using a robust maximum likelihood estimator in Mplus, version 7.0 (Muthén & Muthén, 2017). Participants with at least one available data point were included in the model. The CFA models adequately represented the data, as demonstrated by the root mean square error of approximation (RMSEA) of 0.06, and a confirmatory factor index (CFI) of 0.93. Both factor scores were then extracted and saved for all genotyped participants (*N* = 536). These scores were used in the main analyses so that participants had two overall victimisation scores, one at age 7 and another at age 10 years. The three separate informant scores yielded significant loadings at both 7 and 10 years; 0.60 and 0.72 for peer‐reported victimisation; 0.58 and 0.63 for teacher‐rated victimisation; and 0.37 and 0.43 for self‐reported victimisation, respectively.

### Genotyping

Genotype data were collected from blood or saliva from a subsample of QNTS families at approximately 100 months, including 581 twins (136 MZ twins, 445 DZ twins). All genotyped children were of European descent, with the majority also of white ethnicity (see Table S4). Both twins were included in each twin pair, with family effects modelled in the regression analyses. Data were subject to quality control and imputation, conducted using the 1000 Genomes Phase 3 reference panel. Ancestry components were calculated to determine genetic outliers, of which 10 were used as covariates to control for population stratification. Further information can be found in Appendix S1. Overall, victimisation rates did not differ for youth with or without genotype data (Table S3).

### Polygenic scores

PGSs were derived from publicly available GWAS summary statistics and created using the PRSice software, version 2.2.3 (Euesden et al., 2015). The 10 PGSs were derived from GWASs on the following traits: Major depressive disorder (MDD), attention deficit hyperactivity disorder (ADHD), risk‐taking, body mass index (BMI), intelligence, educational attainment, wellbeing, depressive symptoms, schizophrenia, and extreme BMI. These traits represent all 10 vulnerabilities that were previously identified as predictive of victimisation (Schoeler et al., 2019). Where larger and more recent GWASs were available, these were used instead (see Table S5). There was no overlap between the GWAS samples and the QNTS.

All PGSs were created by combining the number of risk alleles present for each SNP (0, 1, or 2), weighted by their effect estimates reported in the original GWAS. These were used to construct PGSs using imputed genotypes. SNPs with a minor allele frequency (MAF) <0.01, and an imputation quality score <0.8 were removed. Clumping was carried out to remove SNPs in linkage disequilibrium (LD) at *r* ^2^ > 0.10 within a 250‐base pair window. Scores were computed for *p*‐value thresholds between .01 and 1 at .01 increments, generating 99 thresholds in total, as per previous research (Schoeler et al., 2019). An empirical *p‐*value for the best‐fit threshold was generated using permutation (10,000 times). In PRSice, the best‐fit scores are determined using the highest *R* ^2^ estimate from the regression analysis. These analyses controlled for 10 principal components (PCs), and all PGSs were standardised. Correlations between the best‐fit PGSs can be found in the Supporting Information (see Table S6).

## STATISTICAL ANALYSES

### Main analyses

To first replicate previous associations between childhood victimisation and PGSs (Schoeler et al., 2019), linear regression analyses were used to explore whether the PGSs predict our overall measure of self‐reported victimisation in childhood (based on self‐reports at ages 7, 10, and 12 years). We then investigated associations using our mean scores derived from teacher‐ and peer‐reports. Associations between the PGSs and the two aggregated measures of overall childhood victimisation at ages 7 and 10 years were then investigated. We also explored whether associations generalise to victimisation in later adolescence, as based on mean victimisation score. Main analyses were conducted using z‐standardised scores to facilitate comparisons across the different scales. Results from the unstandardised scores can be found in Tables S7 and S8.

For each outcome, associations were first explored for each PGS individually to evaluate their specific contribution (single‐PGS models), we then included significant PGSs to assess their independent contributions (multi‐PGS models). Multicollinearity was not an issue within these models as correlations between PGSs were no larger than 0.35 (Table S6). Every model was adjusted for sex and 10 PCsand run using linear mixed effects models in R studio 3.5.1 (R Core Team, 2018). This was done using the lme4 package (Bates et al., 2014) which allowed us to adjust for the non‐independence of the twin observations (see Appendix S1). Analyses were also corrected for multiple testing using Benjamini‐Hochberg false discovery rate (FDR; Benjamini & Hochberg, 1995) as this allows for the non‐independence of repeated tests (see Appendix S1).

### Longitudinal growth curve model

Longitudinal growth curves were then fit using the ‘lme4’ package in R (Bates et al., 2014). This allowed mixed effects models to explore mean trajectories of victimisation for the entire sample, as well as individual deviations from the mean for each participant. To examine associations between the PGSs and trajectories of victimisation over time, age was first modelled using all self‐reports from 7 to 17 years. To then test whether PGSs had statistically different predictions in childhood versus adolescence, a ‘period’ variable was created. As per the mean composite scores, self‐reports assessed at 7, 10 and 12 years were coded as ‘0’ to represent childhood victimisation, and those assessed at 13, 15 and 17 years were coded as ‘1’ to capture adolescent victimisation. Analyses were also replicated with adolescence coded as ‘0’, and childhood coded as ‘1’ to extract the main effect estimates on adolescent victimisation. All mixed effect models included a main effect of each standardised PGS, an interaction term with age or period, an interaction term between sex and age or period, as well as the 10 PCs. All models also adjusted for the clustering of twin data.

## RESULTS

### Descriptive data

Victimisation scores are presented in Table 1. Overall, significant decreases in victimisation were observed with age, reflected in both teacher‐rated reports, which dropped from 0.26 (SD = 0.37) at age 7 to 0.21 (SD = 0.38) at age 12, and by self‐reported scores, which decreased from 0.71 (SD = 0.52) at age 7 to 0.18 (SD = 0.23) at 17 years. Such findings are consistent with previous research (Oncioiu et al., 2020).

### Associations between PGSs and victimisation in childhood

#### Self‐reported victimisation

**FIGURE 1:**
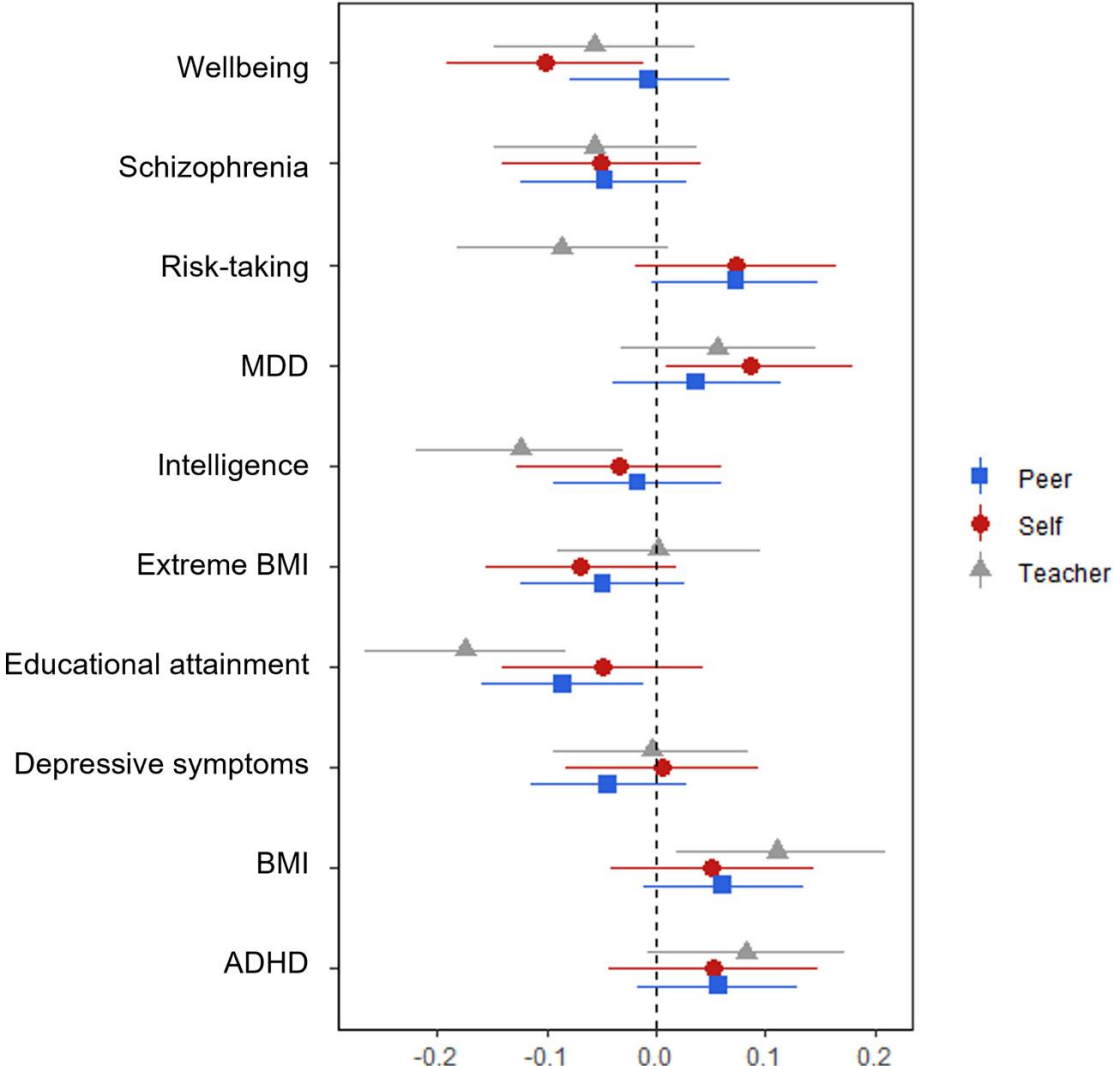
Regression coefficients from single‐PGS (polygenic score) models predicting victimisation using either self‐, teacher‐ or peer‐reports. Associations between teacher‐reported victimisation and the intelligence PGS and the educational attainment PGS survived after correction for multiple testing

In single‐PGS models predicting the self‐reported childhood composite, we replicated the direction of effect from previous reports for 8 out of 10 PGSs, with two PGSs shown to be predictive of our victimisation measure (see Figure 1; Table 2). These were found using the PGS for wellbeing, which predicted a reduced risk of victimisation, and the PGS for MDD, which predicted an increased risk. Effect sizes for the wellbeing PGS were similar to those found previously (Schoeler et al., 2019), but results for the MDD PGS were attenuated. Associations with the wellbeing PGS remained in the multi‐PGS model (see Table S7), suggesting unique contributions. However, neither of the associations survived after correction for multiple testing.

**TABLE 2:** Associations between PGSs and *z*‐standardised self‐, teacher‐, and peer‐reported childhood victimisation

	Single‐PGS models					
	Self‐reported victimisation^a^		Teacher‐reported victimisation^b^		Peer‐reported victimisation^c^	
PGSs	Coefficient, *β*(95% CI)	*p*	Coefficient, *β*(95% CI)	*p*	Coefficient, *β*(95% CI)	*p*
MDD	.086 (.009, .181)	.05	.053 (−.043, .149)	.27	.037 (−.040, .114)	.35
ADHD	.056 (−.031, .147)	.20	.083 (−.007, .173)	.07	.057 (−.016, .129)	.13
Risk‐taking	.073 (−.018, .166)	.11	−.086 (−.183, .011)	.08	.073 (−.003, .149)	.06
BMI	.051 (−.041, .145)	.28	**.110 (.019, .201)**	**.02**	.061 (−.012, .135)	.10
Intelligence	−.034 (−.128, .060)	.47	**−.124 (−.219, −.030)**	**<.001** ^d^	−.017 (−.093, .060)	.66
Educational attainment	−.049 (−.141, .043)	.29	**−.174 (−.267, −.082)**	**<.001** ^d^	**−.085 (−.159, −.011)**	**.02**
Depressive symptoms	.005 (−.082, .093)	.90	−.004 (−.093, .085)	.93	−.044 (−.115, .028)	.23
Wellbeing	−.101 (−.191, −.011)	**.03**	−.057 (−.149, .035)	.22	−.006 (−.079, .067)	.87
Schizophrenia	−.050 (−.141, .041)	.28	−.056 (−.149, .038)	.24	−.047 (−.123, .028)	.21
Extreme BMI	−.069 (−.155, −.018)	.12	.002 (−.091, .096)	.96	−.049 (−.123, .026)	.20

*Note*: Analyses based on linear mixed effects model, controlling for sex and 10 PCs. Associations in bold represent those reaching significance prior to adjustment for multiple testing.

^a^ Based on mean composite of scores from 7, 10 and 12 years.

^b^ Based on mean composite of scores from 7, 10 and 12 years.

^c^ Based on mean composite of scores from 7 to 10 years.

^d^ FDR.

#### Teacher‐reported victimisation

Analyses using teacher‐reported victimisation revealed three novel associations compared to our self‐report measure. These were found using PGSs for BMI, intelligence, and educational attainment (Table 2). The PGSs for intelligence and educational attainment both survived correction for multiple testing, predicting a −0.124 (95% CI = −0.219, −0.030) and a −0.174 (95% CI = −0.267, −0.082) reduction in victimisation for a standard deviation increase in PGS, respectively. The educational attainment PGS also demonstrated an independent contribution in the multi‐PGS model, predicting a −0.040 (95% CI = −0.071, −0.010) decline in victimisation (Table S7).

#### Peer‐reported victimisation

When investigating predictions using our peer‐reported composite, associations were found using the PGS for educational attainment only (see Figure 1). This association did not survive after FDR correction.

#### Overall victimisation

Results using the aggregate, multi‐informant victimisation scores at ages 7 and 10 can be found in Table S9. PGSs associated with overall victimisation at both time points included those for BMI and educational attainment, with educational attainment surviving after correction for multiple testing. When entered into multi‐PGS models, the PGS for educational attainment remained associated with both outcomes.

### Associations between PGSs and self‐reported victimisation in adolescence

With respect to the prediction of self‐reported victimisation in adolescence, some associations found for self‐reported childhood victimisation were replicated, including the PGSs for MDD (see Table S10). We also observed associations with PGSs that did not predict victimisation in childhood. These were found using PGSs for BMI, educational attainment, and extreme BMI. No associations, however, remained after FDR correction, and no independent effects were found in the multi‐PGS model.

#### Longitudinal growth curve models

**FIGURE 2:**
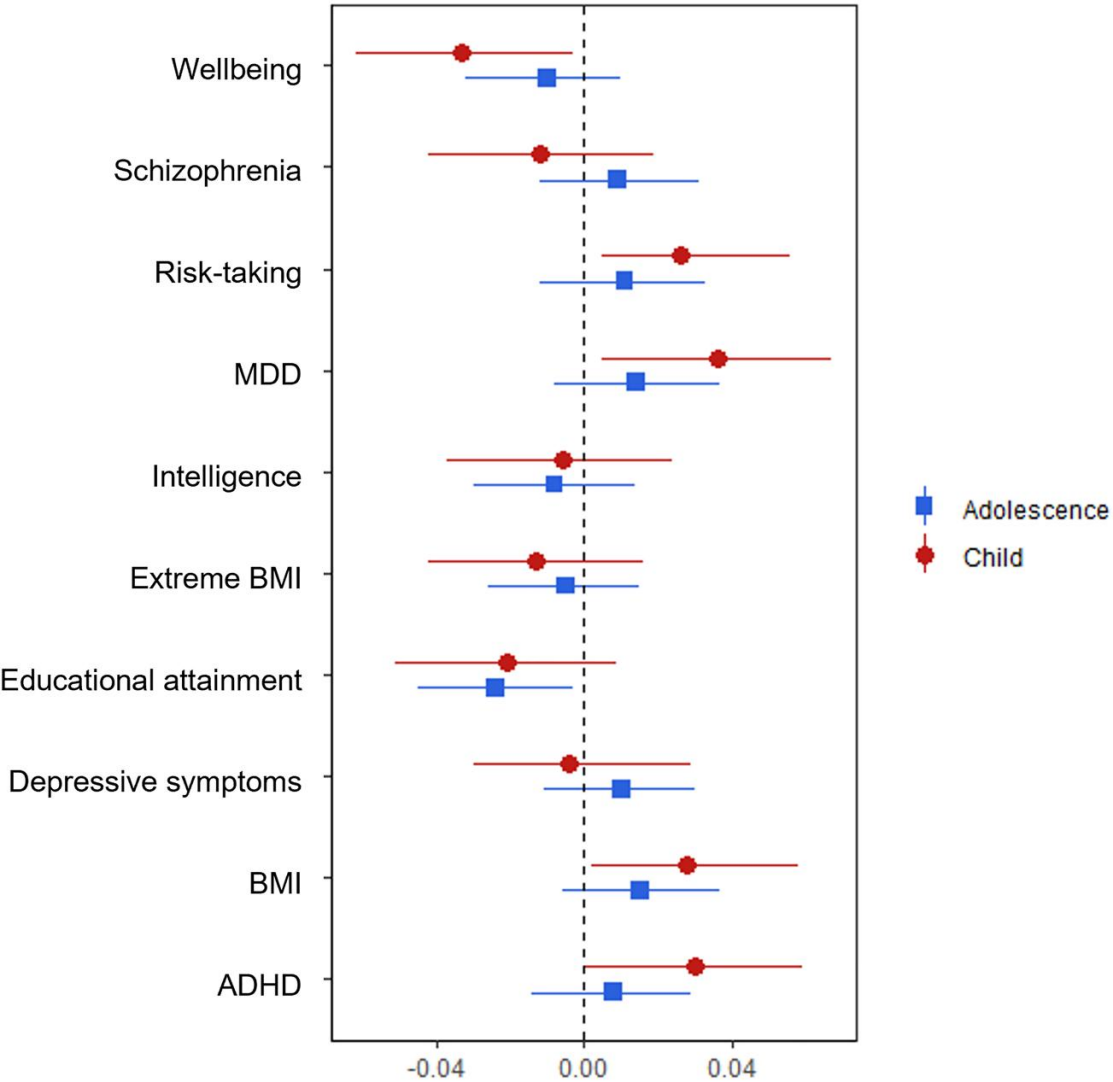
Regression coefficients from single‐PGS (polygenic score) growth‐curve models predicting trajectories in self‐reported victimisation in childhood (7, 10 and 12 years) and adolescence (13, 15 and 17 years)

The first set of growth‐curve analyses revealed that the MDD PGS was associated with levels of victimisation at the intercept (see Table S11), but no PGSs predicted changes in victimisation over time. When investigating whether associations between the PGSs and victimisation differed in childhood versus adolescence, findings revealed that childhood victimisation was predicted by PGSs related to MDD, ADHD, and wellbeing, while adolescent victimisation was associated with the educational attainment PGS (see Figure 2). However, these changes did not differ statistically (Table 3).

**TABLE 3:** Associations between PGSs and self‐reported victimisation across two time periods

	Self‐reported childhood victimisation^a^		Self‐reported adolescent victimisation^b^		Difference between time periods
PGSs	Coefficient, *β*(95%,CI)	*p*	Coefficient, *β*(95%,CI)	*p*	*p*
MDD	.036 (.005, .067)	.02	.014 (−.008, .037)	.21	.89
ADHD	.030 (.00, .059)	.05	.008 (−.014,.029)	.47	.13
Risk‐taking	.026 (−.005, .056)	.09	.011 (−.012, .03)	.35	.31
BMI	.028 (−.002, .058)	.07	.015 (−.006, .037)	.17	.41
Intelligence	−.006 (−.037, .024)	.69	−.008 (−.030, .014)	.49	.84
Educational attainment	−.021 (−.051, .009)	.17	−.024 (−.045, −.003)	.03	.76
Depressive symptoms	−.004 (−.030, .029)	.98	.010 (−.011, .030)	.36	.52
Wellbeing	−.033 (−.062, −.003)	.03	−.010 (−.032, .010)	.34	.14
Schizophrenia	−.012 (−.042, .019)	.45	.009 (−.012, .031)	.41	.18
Extreme BMI	−.013 (−.042, −.017)	.36	−.005 (−.026, .015)	.61	.63

*Note*: Analyses based on repeated measures within mixed effects models, controlling for age * PGS, age * sex, and 10 PCs.

^a^ Based on self‐reported victimisation at 7, 1, and 12 years.

^b^ Based on self‐reported victimisation at 13, 15 and 17 years.

## DISCUSSION

The present study examined the extent to which the genetic propension for known correlated phenotypes, as indexed by PGSs, predicts the risk of peer victimisation when using reports beyond the victims in childhood. In particular, we consider whether findings replicate using teacher‐ and peer‐reports in childhood, as well as self‐reports in later adolescence. Our findings confirm the direction of effect for most of the previously reported PGSs (Schoeler et al., 2019) and show that the risk peer victimisation is predicted by PGSs related to MDD, wellbeing, BMI, educational attainment, intelligence, and extreme BMI. We also extend findings to show that while the direction of effect is similar across informants, there are some possible differences in the size of the associations between the PGSs and different informant report. We also identified some unique genetic liabilities associated with peer victimisation in later adolescence. However, these age differences were not statistically confirmed in mixed effect models exploring trajectories across time.

One of the most consistent findings from our study concerned the PGS for educational attainment. Findings across informants and developmental periods revealed that individuals genetically inclined to complete more years of schooling were at a reduced risk of victimisation. This association may be driven by a number of phenotypic differences that are captured by the PGS for educational attainment, including both cognitive and non‐cognitive factors (Demange et al., 2021).

It is possible that findings reflect the higher cognitive abilities of those at a lower risk of victimisation (Verlinden et al., 2014). While the PGS for intelligence in our study was not as consistently associated with victimisation, this may be attributed to power differences between the GWASs used. The negative relationship between victimisation and educational attainment may also be driven by other factors that covary with its PGS. These include personality traits like extraversion and agreeableness, both of which have been associated with the non‐cognitive component of educational attainment (Demange et al., 2021). Such traits may reduce the risk of victimisation by allowing individuals to foster healthy relationships (Selfhout et al., 2010).

The findings could also reflect differences in family socioeconomic status (SES) and parental education. Both play an important role in shaping a child's academic trajectory, with research indicating genetic and environmental influences of SES on educational attainment (Wang et al., 2021). Victims of bullying are less likely to come from advantaged backgrounds (Tippett & Wolke, 2014), meaning associations between victimisation and educational attainment may be confounded by SES. It is possible that SES increases the risk of victimisation and the number of years of schooling completed, without schooling directly influencing victimisation. Larger studies should test this and explore a possible gene‐by‐environment correlation by comparing polygenic predictions both within‐ and between‐family members (Selzam et al., 2019). Such a design would provide insight into possible paths by which genetic dispositions may influence vulnerability to peer victimisation.

Beyond educational attainment, our study also replicated other associations previously reported (Schoeler et al., 2019). However, findings revealed that some vulnerabilities may be more pronounced depending on the informant. Self‐reported victimisation in both childhood and adolescence was predicted by PGSs associated with mental health, whereas teacher‐ and peer‐reports were predicted by PGSs related to physical and cognitive‐related traits. While not all associations remained after adjustment for multiple testing, it is possible that there may be small differences due to the distinct aspects of victimisation captured by each informant. Self‐reports of victimisation are more highly related to internalising problems than peer‐reports (Bouman et al., 2012). This is because self‐reports tap into subjective appraisals of the self and others, including the victimisation experience, which map onto intrapersonal indicators of maladjustment like depression (Ladd et al., 2002). Peer‐ and teacher‐reports on the other hand, are more likely to capture popularity and social reputation, and are thus more likely to associate with external markers of maladjustment (Ladd et al., 2002). This may account for the link between such reports and genetic proxies of educational attainment and BMI.

Our study emphasised the value of using multiple informants to gain deeper insight into predictors of peer victimisation. It also highlighted the benefit of combing such reports into an aggregated composite. Findings based on our multi‐informant measure produced the largest estimates across our victimisation outcomes, with associations also surviving FDR correction. Such associations are likely to reflect more robust relations compared to single informant measures because potential measurement error is reduced by considering all three perspectives. One downfall, however, is that by extracting scores based on common perspectives, we minimise the ability to explore distinct viewpoints. Thus, future studies should carefully consider how informant reports are used. This decision should depend on the research goals and expectations of convergence (Ladd et al., 2002). The low correlations between informants in our study, in addition to the varying PGS associations, particularly for the risk‐taking PGS which showed opposite effects for self‐ and teacher‐reports, imply that investigating reports as both separate and combined measures may be necessary to capture the complexity of peer victimisation and its associated risk factors. Findings also suggest that research into the mental health outcomes associated with victimisation may benefit from understanding more about the subjective experience of the victim.

Finally, in addition to showing how informant reports can strengthen and complement our understanding of victimisation, we also revealed subtle differences in predictors of victimisation with age. While some vulnerabilities, such as the PGS for depression, were associated with mean victimisation scores in both childhood and later adolescence, some were unique to each time point. When investigating whether differences were statistically different using mixed effect models exploring trajectories, however, findings revealed that while a genetic risk to depression may be a more important predictor of victimisation trajectories in childhood, effects are not significantly larger than those in adolescence. This implies that although modest, the role of a genetic propensity for depression in victimisation is stable over time.

### Limitations

It is important that the findings on age differences are interpreted with some caution given the limited sample size (see Appendix S1). Previous research has shown that the predictive accuracy of PGSs varies with age (Mostafavi et al., 2020). Thus, it is possible that differences in polygenic predictors of victimisation across age reflect greater predictive power of PGSs based on GWASs of adults. Another limitation related to our sample size is that some PGSs likely had more power than others due to being based on larger discovery GWASs. Such differences may explain why the most consistent associations were found with educational attainment as this PGS was based on a GWAS of over 1 million participants from various countries (Lee et al., 2018). Genetic variants captured by this PGS may be more robust to environmental differences in educational institutions across countries, generating more power to detect subtle effects across different informants. Power issues likely also explain why many of the PGSs were only associated with exposures under less stringent conditions (i.e. prior to correction for multiple testing), or not associated at all. For instance, it is possible that the variability in BMI within the current study was not diverse enough to detect associations with the PGS for extreme BMI. It is therefore crucial that larger studies based on well‐powered GWASs attempt to replicate the current findings. Such studies should include more diverse populations as the current study is limited in making generalisation beyond individuals of white European ancestry. Using wider samples and larger GWASs may lead to new and more robust predictors of victimisation.

Other limitations of the current study are that our sample of twins may restrict the generalisability to singletons. It is possible that having a cotwin offers some protection from victimisation, either physically by intervening, or by providing unique social support. Family effects were adjusted for using linear mixed effects models, and peer nominations were standardized within classrooms to allow comparisons with other children. Nevertheless, the use of twin data may be one explanation why our results differ to Schoeler et al. (2019).

Finally, our findings must be considered in relation to the different measures used to assess victimisation. The factor analysis scores were only based on three informant reports, and our peer nomination measure did not include questions about the frequency of victimisation. Some students may therefore have incorrectly nominated peers for infrequent or minor acts of teasing. When compared to the standardised teacher‐ratings, the degree of variation in the peer‐reports was ssimilar, however, information about frequency is crucial to understanding dose‐response relationships and detecting those most at risk. In addition, unlike self‐reports, which covered both direct and relational forms of victimisation, teacher and peer assessments focused on direct forms. Differences between the informant reports and vulnerabilities may thus reflect distinct predictors of overt and relational types of victimisation rather than unique perspectives. Future research should attempt to compare different forms of victimisation using multiple informants to explore potential underlying differences.

## CONCLUSION

Overall, our findings implicate some pre‐existing genetic vulnerabilities as risk factors for victimisation in childhood and adolescence. In particular, genetic proxies associated with educational attainment. Further research should explore this finding further to understand how, and in which contexts, genetic dispositions may increase or decrease vulnerability to peer victimisation. This will be crucial to developing more targeted prevention strategies.

## CONFLICT OF INTEREST

The authors have declared that they have no competing or potential conflicts of interest.

## ETHICAL CONSIDERATION

Parental and child informed consent was obtained from the ethics review board at Université Laval, Quebec.

## AUTHOR CONTRIBUTIONS

**Jessica Armitage:** Conceptualization; Formal analysis; Investigation; Methodology; Project administration; Visualization; Writing – original draft; Writing – review & editing. **Geneviève Morneau‐Vaillancourt:** Conceptualization; Formal analysis; Writing – review & editing. **Jean‐Baptiste Pingault:** Conceptualization; Writing – review & editing. **Till Andlauer:** Data curation; Formal analysis. **Stéphane Paquin:** Conceptualization; Writing – review & editing. **Stéphanie Langevin:** Methodology. **Mara Brendgen:** Writing – review & editing. **Jean Séguin:** Conceptualization. **Guy Rouleau:** Conceptualization. **Michel Boivin:** Conceptualization; Investigation Methodology; Writing – review & editing.

## Supporting information

Supporting Information S1

Supporting Information S2

## Data Availability

The datasets analysed during the current study are not publicly available as the informed consent obtained from QNTS participants does not allow data to be made freely available through any third party maintained public repository. However, data used for this submission can be made available on request, see http://www.gripinfo.ca/grip/public/www/Etudes/en/dadprocedures.asp for more information. The study website also contains details of all the data that are available (https://maelstrom‐research.org/study/ejnq).
