# Supplementary material for: A multi‐informant and multi‐polygenic approach to understanding predictors of peer victimisation in childhood and adolescence
Source: JCPP Adv. 2022 Feb 23;2(1):e12063. doi: 10.1002/jcv2.12063 (PMC10242974; doi:10.1002/jcv2.12063)
Supplement: Supplementary file 1 — Supporting Information S1 [file JCV2-2-e12063-s002.docx]

**Appendix S1: Supplementary Methods**

**A multi-informant and multi-polygenic approach to understanding predictors of peer victimisation in childhood and adolescence.**

Jessica M Armitage^1^, Geneviève Morneau-Vaillancourt, Jean-Baptiste Pingault, Till F. M. Andlauer, Stéphane Paquin, Stéphanie Langevin, Mara Brendgen, Ginette Dionne, Jean Séguin, Guy Rouleau, Frank Vitaro, Isabelle Ouellet-Morin, & Michel Boivin

^1^School of Psychological Science, University of Bristol, United Kingdom

**Sample information**

Zygosity in the QNTS was established when the twins were 5 and 18 months old using a shortened version of the Zygosity Questionnaire for Young Twins (Goldsmith, 1991). Similarity between the twins was concordant with genotyping among 91.9% of cases at 5 months and 93.8% at 18 months (Forget, Dubois, Pérusse, Turecki, Girard, Billette & Rouleau et al., 2003; see Boivin et al., 2019 for details). These estimates are similar to those of other twin studies (Magnusson, Almqvist, Rahman, Ganna, Viktorin & Walum et al., 2013).

**Genotyping procedure: Quality control and imputation**

Genotype data were collected from blood or saliva from a subsample of QNTS families, including 581 twins (136 MZ twins, 445 DZ twins) who were approximately 100 months old. Genotyping was performed at Genome Quebec in Canada, using Illumina’s Psych array Beadchip and carried out in two waves. The first wave was conducted at approximately 100 months old, and the second wave was at 19 years. This second wave resulted in an additional 328 twins (including 38 MZ twins).

Data were subject to quality control, which was conducted in PLINK v1.90b5.3, PLINK v1.90b6.7 (Chang et al., 2015), and R v3.4.3. During quality control, variants with a minor allele frequency (MAF) of <0.01, a SNP genotyping rate of <0.98, and an individual call rate of <0.98 were removed. Variants were also removed if there was evidence of violations of Hardy-Weinberg equilibrium (HWE, p<1x10^-6^). Individuals were also excluded if there were mismatches between genetic and phenotypic sex, or if there was genetic duplication, cryptic relatedness, minimal or excessive heterozygosity, or a potential Klinefelter syndrome diagnosis. After these exclusions, the dataset contained information relating to 849 individuals and 225,119 variants. These data were subject to imputation, which was conducted using SHAPEIT v2 (r837) (Delaneau, Zagury & Marchini, 2013), IMPUTE2 v2.3.2 (Howie, Donnelly & Marchini, 2009), and the 1000 Genomes Phase 3 reference panel. After imputation, variants with a MAF <1%, an HWE test *p*<1×10^-6^, and an INFO metric <0.8 were removed, resulting in a final dataset containing 8,407,807 variants.

# **Calculation of ancestry component (principal components)**

Ancestry components were calculated to determine genetic outliers. Pre-imputation genotype data were used, with additional variant filtering steps. These included the removal of variants with a MAF <0.05 or HWE *p*‑value <10^‑3^, removal of variants mapping to the extended MHC region (chromosome 6, 25-35 Mbp) or to a typical inversion site on chromosome 8 (7‑13 Mbp), and linkage disequilibrium (LD) pruning (command --indep-pairwise 200 100 0.2). Next, the pairwise identity-by-state (IBS) matrix of all individuals was calculated using the command ‑‑genome on the filtered genotype data. Multidimensional scaling (MDS) analysis was performed on the IBS matrix using the eigendecomposition-based algorithm in PLINK v1.90b5.2. One twin per pair was selected at random for PC analysis. Loadings were then copied among the co‐twin to compute PC values for the full sample.

**Polygenic model**

Linear regression analyses were conducted using the lme4 package in R studio. This enabled all models to adjust for clustering within families using the following formula:

res3b = lmer(self_report_childood_vic ~ 1 + MDD_PRS + sexe + cc1 + cc2 + cc3 +cc4 +cc5 + cc6 + cc7 + cc8 +cc9 + cc10 + (1 | nofamill), data = data)

**Benjamini-Hochberg False Discovery Rate**

The Benjamini-Hochberg False Discovery Rate (FDR) procedure was used to correct our analyses for multiple testing. During this method, the corresponding p-values are ranked, with 1 reflecting the smallest p-value. The Benjamini-Hochberg critical value is then calculated using (i/m)*Q. Here, i refers to the p-value rank, m is the total number of tests and Q is the false discovery rate (we used 0.05). The total number of tests adjusted for in the present study was 60. This reflects the 10 PRS regressions that were conducted for each outcome (6 outcomes in total: self-reported childhood composite, teacher-reported childhood composite, peer-reported childhood composite, overall victimisation at age 7, overall victimisation at age 10, self-reported adolescent victimisation). After calculating the Benjamini-Hochberg critical value, we infer those that are robust to multiple testing by taking the largest p-value that is smaller than its corresponding Benjamini-Hochberg critical value. All p-values ranked before this are also deemed significant.

**References**

Boivin, M., Brendgen, M., Dionne, G., Ouellet-Morin, I., Dubois, L., & Pérusse, D., et al. (2019). The Quebec Newborn Twin Study at 21. *Twin Research and Human Genetics*, 1-7.

Chang, C. C., Chow, C. C., Tellier, L. C., Vattikuti, S., Purcell, S. M., & Lee, J. J. (2015). Second-generation PLINK: rising to the challenge of larger and richer datasets. *GigaScience*, *4*, 7.

Delaneau, O., Zagury, J.-F., & Marchini, J. L. (2013). Improved whole-chromosome phasing for disease and population genetic studies. *Nature Methods2*, *10*, 5–6.

Forget-Dubois, N., Pérusse, D., Turecki, G., Girard, A., Billette, J.-M., Rouleau, G., et al. (2003). Diagnosing zygosity in infant twins: Physical similarity, genotyping, and chorionicity. *Twin Research, 6,* 479-485.

Goldsmith, H. H. (1991). A zygosity questionnaire for young twins: A research note. *Behavior Genetics, 21,* 257-269.

Green P., & MacLeod, C. J. (2015). SIMR: an R package for power analysis of generalized linear mixed models by simulation. *Methods in Ecology and Evolution, 7*(4), 493-498.

Howie, B. N., Donnelly, P., & Marchini, J. (2009). A flexible and accurate genotype imputation method for the next generation of genome-wide association studies. *PLoS Genetics*, *5*, e100052.

Magnusson, P. K. E., Almqvist, C., Rahman, I., Ganna, A., Viktorin, A., & Walum, H., et al. (2013). The Swedish Twin Registry: Establishment of a biobank and other recent developments. *Twin Research Human Genetics, 16*(1)*,* 317-329.
