# Supplementary material for: A multi‐informant and multi‐polygenic approach to understanding predictors of peer victimisation in childhood and adolescence
Source: JCPP Adv. 2022 Feb 23;2(1):e12063. doi: 10.1002/jcv2.12063 (PMC10242974; doi:10.1002/jcv2.12063)
Supplement: Supplementary file 2 — Supporting Information S2 [file JCV2-2-e12063-s001.docx]

**Supporting Information: Tables and Figures**

**A multi-informant and multi-polygenic approach to understanding predictors of peer victimisation in childhood and adolescence.**

Jessica M Armitage^1^, Geneviève Morneau-Vaillancourt, Jean-Baptiste Pingault, Till F. M. Andlauer, Stéphane Paquin, Stéphanie Langevin, Mara Brendgen, Ginette Dionne, Jean Séguin, Guy Rouleau, Frank Vitaro, Isabelle Ouellet-Morin, & Michel Boivin

^1^School of Psychological Science, University of Bristol, United Kingdom

**Index of Supplementary Tables and Figures**

1. **Table S1:** Socio-demographics comparisons of participants with complete and missing victimisation data at 7 and 17 years
2. **Table S2:** Victimisation item responses
3. **Table S3:** Correlations between different informant reports of victimisation
4. **Table S4:** Socio-demographics comparisons of participants with complete and missing data
5. **Table S5:** GWAS information and power analyses for PGSs using the avengeme software
6. **Table S6:** Genetic correlations between PGSs
7. **Table S7:** Associations between PGSs and unstandardised self-, teacher-, and peer-reported childhood victimisation measures
8. **Table S8:** Associations between PGSs and unstandardised self-reported adolescent victimisation
9. **Table S9:** Associations between PGSs and the factor analysis scores at 7 and 10 years
10. **Table S10:** Associations between PGSs and z-standardised self-reported victimisation in adolescence
11. **Table S11:** Associations between PGSs and self-reported victimisation across age
12. **Figure S1:** Two-factor confirmatory analysis

| **Table S1:** Socio-demographics comparisons of participants with complete and missing victimisation data at 7 and 17 years | | | |  |
| --- | --- | --- | --- | --- |
|  | **Victimisation responders aged 7**  **(n=527)** | **Victimisation responders aged 17 (n=429)** | **Victimisation responders missing^c^ (n=129)** | |
| **Phenotypic comparisons** |  |  |  | |
| Victimisation aged 7 (mean,SD) | 0.71 (0.52) | 0.69 (0.50) | - | |
| White (%) | 99.6 | 100 | 98.3 | |
| White mother (%) | 99.6 | 100 | 98.3 | |
| White father (%) | 99.6 | 100 | 98.3 | |
| Canadian ancestors (%) | 66.1 | 63.1 | 73.6 | |
| Household income above £50,000 (%) | 51.8 | 53.1 | 46.2 | |
| Mother has University degree or higher qualification (%) | 27.0 | 28.1 | 26.1 | |
| Father has University degree or higher qualification (%) | 32.5 | 33.7 | 25.2 | |
| **Genetic** **comparisons** |  |  |  | |
| MDD-PGS (% 1 SD over mean) | 15.6 | 17.5 | 10.1 | |
| ADHD-PGS (% 1 SD over mean) | 15.4 | 17.9 | 18.6 | |
| Risk-taking-PGS (% 1 SD over mean) | 16.3 | 17.2 | 14.7 | |
| BMI-PGS (% 1 SD over mean) | 15.6 | 15.9 | 17.8 | |
| Intelligence-PGS (% 1 SD over mean) | 17.3 | 18.2 | 15.5 | |
| Educational attainment-PGS (% 1 SD over mean) | 15.4 | 14.0 | 18.6 | |
| Depressive symptoms-PGS (% 1 SD over mean) | 17.6 | 12.4 | 20.1 | |
| Wellbeing-PGS (% 1 SD over mean) | 15.4 | 15.9 | 18.6 | |
| Schizophrenia-PGS (% 1 SD over mean) | 15.0 | 17.2 | 15.5 | |
| Extreme-BMI- PGS (% 1 SD over mean) | 16.3 | 15.4 | 18.6 | |
| Note:  ^a^ Individuals with genotype and self-reported victimisation data at 7 years.  ^b^ Individuals with genotype and self-reported victimisation data at 17 years.  ^c^ Individuals with genotype and self-reported victimisation data at 7 years but not 17 years. | | | |  |

| **Table S2:** Victimisation item responses | | | | | | | | | | | | | | | | | | | | | | | | | | | | | | | | | | | |
| --- | --- | --- | --- | --- | --- | --- | --- | --- | --- | --- | --- | --- | --- | --- | --- | --- | --- | --- | --- | --- | --- | --- | --- | --- | --- | --- | --- | --- | --- | --- | --- | --- | --- | --- | --- |
|  | ***Self-reported victimisation responses, n (%)*** | | | | | | | | | | | | | | | | | | | | | | | | | | | | | | | | | | |
|  | | | **7 years** | | | |  | **10 years** | | | | | | **13 years** | | | | | | **15 years** | | | | | | | | **17 years** | | | | | | | |
| **Item** | | | Never | | Sometimes | | Often | Never | | Sometimes | Often | | | Never | | Sometimes | Often | | | Never | | | Sometimes | | Often | | | Never | | | Sometimes | | | Often |  |
| Another student has yelled mean things | | | 328  (35.0) | | 234 (25.0) | | 374 (40.0) | 205  (24.3) | | 393  (46.7) | 244  (29.0) | | | 322  (39.6) | | 361  (44.3) | 131  (16.1) | | | 456  (57.8) | | | 259  (32.8) | | 74  (9.4) | | | 538 (67.2) | | | | 218  (27.2) | | 45 (5.6) |  |
| Another student has said bad things behind your back | | | 476  (51.0) | | 226  (24.2) | | 231  (24.8) | 269  (32.0) | | 381  (45.3) | 190  (22.7) | | | 366  (45.1) | | 337  (41.6) | 108  (13.1) | | | 503  (63.7) | | | 222  (28.1) | | 65  (8.2) | | | 520 (65.1) | | | | 222  (27.8) | | 57 (7.1) |  |
| Another student prevented you from playing in the group | | | 389  (41.6) | | 293  (31.4) | | 252  (27.0) | 348  (41.5) | | 368  (43.9) | 122  (14.6) | | | 658  (81.1) | | 121  (14.9) | 32  (4.0) | | | 729  (92.2) | | | 55  (7.0) | | 7  (0.8) | | | 745 (92.9) | | | | 44  (5.5) | | 13 (1.6) |  |
| Another student has pushed, hit, or kicked you | | | 342  (36.8) | | 272  (29.2) | | 316  (34.0) | 291  (34.7) | | 380  (45.3) | 168  (20.0) | | | 580  (71.3) | | 185  (22.8) | 48  (5.9) | | | 715  (90.5) | | | 67  (8.4) | | 9  (1.1) | | | 744 (0.93) | | | | 48  (0.06) | | 8 (0.01) |  |
| Another student has forced you to do something/give them something you did not want to | | | 495  (53.1) | | 185  (19.8) | | 253  (27.1) | 681  (81.3) | | 131  (15.6) | *26*  *(3.1)* | | | 780  (95.9) | | 27  (3.3) | 6  (0.8) | | | 777  (98.2) | | | 13  (1.7) | | 1  (0.1) | | | 795 (99.2) | | | | 7  (0.7) | | 1  (0.1) |  |
|  | ***Teacher-reported victimisation responses, n (%)*** | | | | | | | | | | | | | | | | | | | | | | | | | | | | | | | | | | |
|  | | **7 years** | | | | | | | **10 years** | | | | | **12 years** | | | | |  | | |  | | | | |  |  | |  | | | |  |  |
| **Item** | | Never | | Sometimes | | Often | | | Never | Sometimes | | Often | Never | | Sometimes | | | Often | | |  | | |  | |  | | |  |  | | |  | |  |
| The student was made fun of by other children | | 632 (76.0) | | 188  (22.6) | | 12  (1.4) | | | 576  (74.1) | 182  (23.4) | | 19  (2.5) | 491  (78.1) | | 116  (18.4) | | | 22  (3.5) | | |  | | |  | |  | | |  |  | | |  | |  |
| The student was hit or pushed by others | | 529 (63.7) | | 283  (34.1) | | 20  (2.2) | | | 589  (75.7) | 179  (23.0) | | 10  (1.3) | 538  (85.5) | | 83  (13.2) | | | 8  (1.3) | | |  | | |  | |  | | |  |  | | |  | |  |
| The student was called names by others | | 691 (82.9) | | 133  (15.9) | | 11  (1.2) | | | 583  (75.1) | 172  (22.1) | | 22  (2.8) | 514  (81.7) | | 95  (15.1) | | | 19  (3.2) | | |  | | |  | |  | | |  |  | | |  | |  |
|  | | | | | | | | | | | | | | | | | | | | | | | | | | | | | | | | | | |  |

| **Table S3:** Correlations between different informant reports of victimisation | | | | | | | | | | | | | |  | |  |  | | |  | |  |  |
| --- | --- | --- | --- | --- | --- | --- | --- | --- | --- | --- | --- | --- | --- | --- | --- | --- | --- | --- | --- | --- | --- | --- | --- |
| **Variables** | **Correlation matrix** | | | | | | | | | | | | | | |  |  | | |  | |  |  |
|  | **1** | **2** | **3** | **4** | **5** | **6** | **7** | **8** | **9** | **10** | **11** | **12** | **13** | | **14** | | | **15** | **16** | | **17** | |  |
| ***Age 7*** |  |  |  |  |  |  |  |  |  |  |  |  |  | |  | | |  |  | |  | |  |
| 1. Self-reported | 1 | .20*** | 24*** | .49*** | .25*** | .11*** | .15*** | .32*** | .15*** | .11* | .11*** | .01 | .05 | | .75*** | | | .19*** | .23*** | | .07 | |  |
| 1. Teacher-reported |  | 1 | 35*** | .76*** | .12** | .29*** | .30*** | .51*** | .20*** | .26*** | .21*** | .16*** | .08* | | .23*** | | | .73*** | .37*** | | .22*** | |  |
| 1. Peer-reported |  |  | 1 | .78*** | .20*** | .17*** | .29*** | .50*** | .14*** | .28*** | .22*** | .11* | .14** | | .34*** | | | .36*** | .87*** | | .22*** | |  |
| 1. Overall victimisation^a^ |  |  |  | 1 | .39*** | .53*** | .62*** | .84*** | .33*** | .39*** | .33*** | .18*** | .15*** | | .57*** | | | .74*** | .81*** | | .31*** | |  |
| ***Age 10*** | |  |  |  |  |  |  |  |  |  |  |  |  | |  | | |  |  | |  | |  |
| 1. Self-reported |  |  |  |  | 1 | .29*** | .20*** | .53*** | .40*** | .22*** | .30*** | .19*** | .12** | | .76*** | | | .29*** | .27*** | | .30*** | |  |
| 1. Teacher-reported |  |  |  |  |  | 1 | .17*** | .77*** | .25*** | .38*** | .21*** | .15*** | .12*** | | .30*** | | | .80*** | .39*** | | .23*** | |  |
| 1. Peer-reported |  |  |  |  |  |  | 1 | .87*** | .33*** | .31*** | .29*** | .18*** | .10* | | .34*** | | | .48*** | .85*** | | .25*** | |  |
| 1. Overall victimisation^a^ |  |  |  |  |  |  |  | 1 | .40*** | .44*** | .35*** | .22*** | .16*** | | .56*** | | | .76*** | .78*** | | .34*** | |  |
| ***Age 12*** |  |  |  |  |  |  |  |  |  |  |  |  |  | |  | | |  |  | |  | |  |
| 1. Self-reported |  |  |  |  |  |  |  |  | 1 | .34*** | .58*** | .39*** | .31*** | | .67*** | | | .34*** | .27*** | | .55*** | |  |
| 1. Teacher-reported   ***Age 13*** |  |  |  |  |  |  |  |  |  | 1 | .33*** | .24*** | .15*** | | .29*** | | | .77*** | .33*** | | .31*** | |  |
| 1. Self-reported   ***Age 15*** |  |  |  |  |  |  |  |  |  |  | 1 | .41*** | .34*** | | .43*** | | | .34*** | .31*** | | .83*** | |  |
| 1. Self-reported |  |  |  |  |  |  |  |  |  |  |  | 1 | .42*** | | .25*** | | | .24*** | .17*** | | .77*** | |  |
| ***Age 17*** |  |  |  |  |  |  |  |  |  |  |  |  |  | |  | | |  |  | |  | |  |
| 1. Self-reported |  |  |  |  |  |  |  |  |  |  |  |  | 1 | | .22*** | | | .16*** | .13*** | | .72*** | |  |
| ***Mean scores across ages*** | |  |  |  |  |  |  |  |  |  |  |  |  | |  | | |  |  | |  | |  |
| 1. Self-reported childhood^b^ |  |  |  |  |  |  |  |  |  |  |  |  |  | | 1 | | | 0.36*** | 0.36*** | | 0.40*** | |  |
| 1. Teacher-reported childhood^c^ |  |  |  |  |  |  |  |  |  |  |  |  |  | |  | | | 1 | 0.48*** | | 0.35*** | |  |
| 1. Peer-reported childhood^d^ |  |  |  |  |  |  |  |  |  |  |  |  |  | |  | | |  | 1 | | 0.29*** | |  |
| 1. Self-reported adolescence^e^ |  |  |  |  |  |  |  |  |  |  |  |  |  | |  | | |  |  | | 1 | |  |
| Note:  ^a^ Overall victimisation represents factor analysis scores based on peer-, teacher, and self-reports.  ^b^ Self-reported childhood composite based on assessments from 7, 10, and 13 years.  ^c^ Teacher composite based on assessments from 7, 10, and 12 years.  ^d^ Peer composite based on assessments from 7 and 10 years.  ^e^ Self-reported adolescent composite based on assessments from 13, 15, and 17 years. | | | | | | | | | | | | | | | | | | | | | | |  |

| **Table S4:** Socio-demographics comparisons of participants with complete and missing genotype data | | | | | |  |
| --- | --- | --- | --- | --- | --- | --- |
|  | **Complete cases**^a^  **(n=527)** | **Victimisation responders**^b^ **(n=939)** | **Victimisation responders with missing genotype (n=412)** | **QNTS Sample (n=1260)**^c^ | **Missing QNTS Sample**^d^  **(n=733)** | |
| White (%) | 99.6 | 90.4 | 78.6 | 86.3 | 77.6 | |
| White mother (%) | 99.6 | 91.6 | 81.1 | 88.1 | 80.1 | |
| White father (%) | 99.6 | 92.2 | 82.6 | 93.2 | 79.7 | |
| Canadian ancestors (%) | 66.1 | 55.7 | 42.1 | 50.3 | 39.8 | |
| Household income above £50,000 (%) | 51.8 | 48.2 | 40.8 | 43.4 | 37.2 | |
| Mother has University degree or higher qualification (%) | 27.0 | 26.4 | 25.6 | 27.9 | 28.5 | |
| Father has University degree or higher qualification (%) | 32.5 | 31.3 | 29.9 | 29.1 | 26.8 | |
| Note:  ^a^ Complete cases represent those with genotype data and data on victimisation at 7 years (Grade 1).  ^b^ Individuals with victimisation data at 7 years but who did not provide genotype data.  ^c^ Core QNTS sample assessed at 5 months.  ^d^ Core QNTS sample not included in complete cases due to absent genotype data or victimisation data. | | | | | |  |

| **Table S5:** GWAS information | | | | |
| --- | --- | --- | --- | --- |
| **Phenotype** | **Reference to GWAS** | **GWAS discovery sample size** | **Year GWAS published** | **Proportion of total variance explained by genetic effects in discovery sample** |
| Major depressive disorder | Howard, D. M., Adams, M. J., Clarke, T., Hafferty, J. D., Gibson, J., & Shirali, M. et al. (2019). Genome-wide meta-analysis of depression identifies 102 independent variants and highlights the importance of the prefrontal brain regions. *Nature Neuroscience, 22,* 343-352. | 807,553 | 2019 | 3.2% |
| ADHD | Demontis, D., Walters, R. K., Martin, J., Mattheisen, M., Als, T. D., & Agerbo, E., et al. (2017). Discovery of the first genome - wide significant risk loci for attention/deficit hyperactivity disorder. *Nature Genetics, 51*, 63-75. | 55,374 | 2017 | 5.5% |
| Risk-taking | Karlsson Linnér, R., Biroli, P., Kong, E., Meddens, S. F. W., & Wedow, R., et al. (2019). Genome-wide association analyses of risk tolerance and risky behaviors in over one million individuals identify hundreds of loci and shared genetic influences. *Nature Genetics, 51,* 245-257 | 939,908 | 2019 | 1.6% |
| BMI | Yengo L, Sidorenko J, Kemper KE, et al. (2018). Meta-analysis of genome-wide association studies for height and body mass index in ~700,000 individuals of European ancestry. bioRxiv, 274654. | 456,426 | 2018 | 6% |
| Intelligence | Savage JE, Jansen PR, Stringer S, et al. (2018). Genome-wide association meta - analysis in 269,867 individuals identifies new genetic and functional links to intelligence. *Nature Genetics, 50*(7), 912 -919. | 269,867 | 2018 | 5.2% |
| Educational attainment | Lee, J. J., Wedow, R., Okbay, A., et al. (2018). Gene discovery and polygenic prediction from a genome -wide association study of educational attainment in 1.1 million individuals. *Nature Genetics, 50*(8),1112-1121. | 1,131,881 | 2018 | 12% |
| Depressive symptoms | Okbay, A., Baselmans, B. M. L., De Neve, J-E., et al. (2016). Genetic variants associated with subjective well-being, depressive symptoms and neuroticism identified through genome-wide analyses. *Nature Genetics, 48*(6), 624-633. | 161,460 | 2016 | 0.5% |
| Wellbeing | Baselmans, B. M. L., Jansen, R., Ip, H. F., van Dongen, J., Abdellaoui, A., van de Weijer, M. P., & Bao, Y., et al. (2019). Multivariate genome-wide analyses of the well-being spectrum. *Nature Genetics, 51,* 445-451. | 2,370,390 | 2019 | 0.94% |
| Schizophrenia | Bipolar Disorder and Schizophrenia Working Group of the Psychiatric Genomics Consortium. (2018). Genomic Dissection of Bipolar Disorder and Schizophrenia, Including 28 Subphenotypes. *Cell 173*, 170501715 | 107,620 | 2018 | 4.26% |
| Extreme BMI | Berndt SI, Gustafsson S, Mägi R, et al. Genome -wide meta -analysis identifies 11 new loci for anthropometric traits and provides insights into genetic architecture. *Nature Genetics, 45*(5),501-512. | 263,407 | 2013 | 6.4% |

| **Table S6:** Genetic correlations between PGSs | | | | | | | |  |  |  |
| --- | --- | --- | --- | --- | --- | --- | --- | --- | --- | --- |
| **Variables** | **Correlation matrix** | | | | | | |  |  |  |
|  | **1** | **2** | **3** | **4** | **5** | **6** | **7** | **8** | **9** | **10** |
|  |  |  |  |  |  |  |  |  |  |  |
| 1. Major depressive disorder | 1 | .14*** | .04 | .10* | -.06 | -.18*** | .26*** | -.48*** | .15*** | .10* |
| 1. ADHD |  | 1 | .02 | .13** | -.12** | -.21*** | .02 | -.06 | .05 | .05 |
| 1. Risk-taking |  |  | 1 | .08* | .00 | .01 | -.05 | -0.0 | .10* | -.01 |
| 1. BMI |  |  |  | 1 | -.09* | -.15*** | .06 | .04 | -.06 | .32*** |
| 1. Intelligence |  |  |  |  | 1 | .35*** | -.10* | .12** | -.06 | -.02 |
| 1. Educational attainment |  |  |  |  |  | 1 | -.14*** | .17*** | .01 | .07 |
| 1. Depressive symptoms |  |  |  |  |  |  | 1 | -.32*** | -.07 | .10* |
| 1. Wellbeing |  |  |  |  |  |  |  | 1 | -.08* | -.12*** |
| 1. Schizophrenia |  |  |  |  |  |  |  |  | 1 | .10* |
| 1. Extreme BMI |  |  |  |  |  |  |  |  |  | 1 |
| *p<0.05, **p<0.01, ***p<0.001.  Note: Correlations based on a sample of individuals with complete genotype data who also completed measures of peer victimisation aged 7 (n=536). | | | | | | | | | | |

| **Table S7:** Associations between PGSs and unstandardised self-, teacher-, and peer-reported childhood victimisation measures | | | | | | | | | | | | | | | | | | | |
| --- | --- | --- | --- | --- | --- | --- | --- | --- | --- | --- | --- | --- | --- | --- | --- | --- | --- | --- | --- |
|  | **Single-PGS models** | | | | | | | | | | | | | | | | | | |
|  | | | Self-reported victimisation^a^ | | | |  | Teacher-reported victimisation^b^ | | | | | |  | | Peer-reported victimisation^c^ | | | |
| PGSs | | | Coefficient, β(95%,CI) | SE | *p* |  |  | Coefficient, β(95%,CI) | SE | | *p* | |  |  | | Coefficient, *β*(95%,CI) | SE | *p* | |
| MDD | | | 0.028 (0.003,0.060) | 0.02 | 0.05 | |  | 0.016 (-0.013,0.045) | | 0.01 | | 0.27 | | |  | 0.037 (-0.040,0.114) | 0.04 | 0.35 |  |
| ADHD | | | 0.019 (-0.010,0.049) | 0.02 | 0.20 | |  | 0.025 (-0.002,0.052) | | 0.01 | | 0.07 | | |  | 0.057 (-0.016,0.129) | 0.04 | 0.13 |  |
| Risk-taking | | | 0.025 (-0.006,0.055) | 0.02 | 0.11 | |  | -0.026 (-0.055,0.003) | | 0.01 | | 0.08 | | |  | 0.073 (-0.003,0.149) | 0.04 | 0.06 |  |
| BMI | | | 0.017 (-0.014,0.048) | 0.02 | 0.28 | |  | 0.033 (0.006,0.061) | | 0.01 | | 0.02 | | |  | 0.061 (-0.012,0.135) | 0.04 | 0.10 |  |
| Intelligence | | | -0.011 (-0.043,0.020) | 0.02 | 0.47 | |  | **-0.038 (-0.066,-0.009)** | | **0.01** | | **<.001†** | | |  | -0.017 (-0.093,0.060) | 0.04 | 0.66 |  |
| Educational attainment | | | -0.016 (-0.047,0.014) | 0.02 | 0.29 | |  | **-0.053 (-0.081,-0.025)** | | **0.01** | | **<.001†** | | |  | -0.085 (-0.159,-0.011) | 0.04 | 0.02 |  |
| Depressive symptoms | | | 0.002 (-0.027,0.031) | 0.02 | 0.90 | |  | -0.001 (-0.028,0.026) | | 0.01 | | 0.93 | | |  | -0.044 (-0.115,0.028) | 0.04 | 0.23 |  |
| Wellbeing | | | -0.034 (-0.064,-0.004) | 0.02 | 0.03 | |  | -0.017 (-0.045,0.011) | | 0.01 | | 0.22 | | |  | -0.006 (-0.079,0.067) | 0.04 | 0.87 |  |
| Schizophrenia | | | -0.017 (-0.047,0.013) | 0.02 | 0.28 | |  | -0.017 (-0.045,0.011) | | 0.01 | | 0.24 | | |  | -0.047 (-0.123,0.028) | 0.04 | 0.21 |  |
| Extreme BMI | | | -0.023 (-0.052,-0.006) | 0.02 | 0.12 | |  | 0.001 (-0.027,0.028) | | 0.01 | | 0.96 | | |  | -0.049 (-0.123,0.026) | 0.04 | 0.20 |  |
|  | | **Multi-PRS model** | | | | | | | | | | | | | | | | | |
| MDD | | | 0.016 (-0.019,0.052) | 0.02 | 0.36 | |  | **-** | | **-** | | **-** | | |  | - | - | - |  |
| Wellbeing | | | -0.033 (-0.068,-0.001) | 0.02 | 0.05 | |  | - | | - | | - | | |  | - | - | - |  |
| Extreme BMI | | | -0.037 (-0.067,-0.008) | 0.02 | 0.01 | |  | - | | - | | - | | |  | - | - | - |  |
| BMI | | | - | - | - | |  | 0.023 (-0.005,0.051) | | 0.02 | | 0.11 | | |  | - | - | - |  |
| Intelligence | | | - | - | - | |  | -0.021 (-0.051,0.009) | | 0.02 | | 0.18 | | |  | - | - | - |  |
| Educational attainment | | | - | - | - | |  | **-0.040 (-0.071, -0.010)** | | **0.02** | | **<0.001** | | |  | - | - | - |  |
| Note: Single-PGS analyses based on linear mixed effects model, controlling for sex and 10 principal components. Multi-PGS analyses for each outcome used the PGSs identified in the single-PRS models. These analyses were also conducted using linear mixed effects model, controlling for sex and 10 principal components. †FDR  ^a^ Based on mean composite of scores from 7, 10, and 12 years.  ^b^ Based on mean composite of scores from 7, 10, and 12 years.  ^c^ Based on mean composite of scores from 7 and 10 years. | | | | | | | | | | | | | | | | | | | |

| **Table S8:** Associations between PGSs and unstandardised self-reported victimisation in adolescence | | | | | | | | |
| --- | --- | --- | --- | --- | --- | --- | --- | --- |
|  | **Single-PGS regression models** | | |  | | **Multi-PGS regression models** | | |
| PGSs | Coefficient, β(95% CI) | SE | *p* | |  | Coefficient, β(95% CI) | SE | *p* |
| MDD | 0.029 (0.007, 0.050) | 0.01 | 0.01 | |  | 0.021 (-0.00, 0.043) | 0.01 | 0.06 |
| ADHD | 0.011 (-0.010, 0.032) | 0.01 | 0.29 | |  | - | - | - |
| Risk-taking | -0.011 (-0.034, 0.012) | 0.01 | 0.33 | |  | - | - | - |
| BMI | 0.024 (0.003, 0.046) | 0.01 | 0.03 | |  | 0.013 (-0.010, 0.036) | 0.01 | 0.27 |
| Intelligence | -0.013 (-0.035, 0.008) | 0.01 | 0.22 | |  | - | - | - |
| Educational attainment | -0.024 (-0.045, -0.003) | 0.01 | 0.02 | |  | -0.021 (-0.043, 0.000) | 0.01 | 0.06 |
| Depressive symptoms | 0.010 (-0.011, 0.031) | 0.01 | 0.37 | |  | - | - | - |
| Wellbeing | -0.019 (-0.041, 0.003) | 0.01 | 0.09 | |  | - | - | - |
| Schizophrenia | 0.017 (-0.005, 0.039) | 0.01 | 0.13 | |  | - | - | - |
| Extreme BMI | 0.022 (0.000, 0.045) | 0.01 | 0.04 | |  | 0.019 (-0.004, 0.043) | 0.01 | 0.11 |
| Note*:* Analyses were conducted using linear mixed effects model, controlling for sex and the first 10 principal components for stratification. | | | | | | | | |

| **Table S9:** Associations between PGSs and the factor analysis scores at 7 and 10 years | | | | | | | | | | |
| --- | --- | --- | --- | --- | --- | --- | --- | --- | --- | --- |
|  | | **Single-PGS models** | | | | | | | |  |
|  | Overall victimisation age 7 | | | | |  | Overall victimisation age 10 | | |  |
| PGSs | Coefficient, β(95%,CI) | | SE | *p* |  | Coefficient, β(95%,CI) | | SE | *p* |  |
| MDD | 0.049 (-0.019,0.116) | | 0.03 | 0.16 |  | 0.046 (-0.020,0.113) | | 0.03 | 0.17 |  |
| ADHD | 0.059 (-0.005,0.123) | | 0.03 | 0.07 |  | 0.059 (-0.005,0.122) | | 0.03 | 0.07 |  |
| Risk-taking | 0.008 (-0.060,0.076) | | 0.03 | 0.82 |  | -0.017 (-0.083,0.050) | | 0.03 | 0.62 |  |
| BMI | 0.066 (0.002,0.130) | | 0.03 | 0.04 |  | 0.065 (0.002,0.128) | | 0.03 | 0.04 |  |
| Intelligence | -0.041 (-0.108,0.027) | | 0.03 | 0.23 |  | -0.051 (-0.117,0.016) | | 0.03 | 0.13 |  |
| Educational attainment | **-0.094 (-0.159,-0.030)** | | **0.03** | **<.001†** |  | **-0.104 (-0.169,-0.039)** | | **0.03** | **<.001†** |  |
| Depressive symptoms | -0.013 (-0.076,0.051) | | 0.03 | 0.69 |  | -0.026 (-0.089,0.037) | | 0.03 | 0.42 |  |
| Wellbeing | -0.028 (-0.092,0.037) | | 0.03 | 0.39 |  | -0.029 (-0.093,0.036) | | 0.03 | 0.38 |  |
| Schizophrenia | -0.051 (-0.117,0.015) | | 0.03 | 0.13 |  | -0.046 (-0.112,0.020) | | 0.03 | 0.17 |  |
| Extreme BMI | -0.037 (-0.103,0.028) | | 0.03 | 0.26 |  | -0.022 (-0.087,0.044) | | 0.03 | 0.52 |  |
|  | **Multi-PGS model** | | | | | | | | |  |
| BMI | 0.061 (-0.002,0.126) | | 0.03 | 0.06 |  | 0.049 (-0.014,0.11) | | 0.04 | 0.13 |  |
| Educational attainment | **-0.089 (-0.154, -0.024)** | | **0.03** | **<0.001** |  | **-0.096 (-0.161, 0.030)** | | **0.04** | **<0.001** |  |
| Note: Single-PGS analyses based on linear mixed effects model, controlling for sex and 10 principal components. Multi-PGS analyses for each outcome used the PGSs identified in the single-PRS models. These analyses were also conducted using linear mixed effects model, controlling for sex and 10 principal components. Factor analysis scores were created at ages 7 and 10 years using self-, teacher-, and peer-reports. †FDR. | | | | | | | | | |  |

| **Table S10:** Associations between PGSs and z-standardised self-reported victimisation in adolescence | | | | | | | | |
| --- | --- | --- | --- | --- | --- | --- | --- | --- |
|  | **Single-PGS regression models** | | |  | | **Multi-PGS regression models** | | |
| PGSs | Coefficient, β(95% CI) | SE | *p* | |  | Coefficient, β(95% CI) | SE | *p* |
| MDD | 0.137 (0.033, 0.243) | 0.05 | 0.01 | |  | 0.102 (-0.004, 0.209) | 0.05 | 0.06 |
| ADHD | 0.055 (-0.046, 0.155) | 0.05 | 0.29 | |  | - | - | - |
| Risk-taking | -0.055 (-0.165, 0.056) | 0.05 | 0.33 | |  | - | - | - |
| BMI | 0.117 (0.013, 0.221) | 0.05 | 0.03 | |  | 0.062 (-0.049, 0.174) | 0.06 | 0.27 |
| Intelligence | -0.065 (-0.169, 0.004) | 0.05 | 0.22 | |  | - | - | - |
| Educational attainment | -0.117 (-0.218, -0.015) | 0.05 | 0.02 | |  | -0.101 (-0.207, 0.004) | 0.05 | 0.06 |
| Depressive symptoms | 0.046 (-0.055, 0.147) | 0.05 | 0.37 | |  | - | - | - |
| Wellbeing | -0.090 (-0.195, 0.016) | 0.05 | 0.09 | |  | - | - | - |
| Schizophrenia | 0.082 (-0.024, 0.187) | 0.05 | 0.13 | |  | - | - | - |
| Extreme BMI | 0.108 (0.002, 0.214) | 0.05 | 0.04 | |  | 0.093 (-0.020, 0.206) | 0.06 | 0.11 |
| Note*:* Analyses were conducted using linear mixed effects model, controlling for sex and the first 10 principal components for stratification. | | | | | | | | |

| Table S11: Associations between PGSs and self-reported victimisation across age | | | | | | | | | | | |
| --- | --- | --- | --- | --- | --- | --- | --- | --- | --- | --- | --- |
|  | **Single-PGS growth-curve models** | | | | | | | | | | |
|  | | Intercept | | | |  | | Slope | | | |
| PGSs | | Coefficient, β(95%,CI) | SE | *p* |  |  | | Coefficient, β(95%,CI) | SE | | *p* |
| MDD | | 0.078 (0.003, 0.157) | 0.04 | 0.04 | |  | -0.004 (-0.010,0.001) | | | 0.003 | 0.13 |
| ADHD | | 0.061 (-0.011, 0.133) | 0.04 | 0.09 | |  | -0.004 (-0.009,0.001) | | | 0.003 | 0.15 |
| Risk-taking | | 0.034 (-0.041, 0.109) | 0.04 | 0.37 | |  | -0.001 (-0.007, 0.004) | | | 0.003 | 0.59 |
| BMI | | 0.057 (-0.015, 0.129) | 0.04 | 0.12 | |  | -0.003 (-0.008, 0.002) | | | 0.003 | 0.27 |
| Intelligence | | -0.014 (-0.088, 0.061) | 0.04 | 0.72 | |  | 0.000 (-0.005, 0.006) | | | 0.003 | 0.82 |
| Educational attainment | | -0.042 (-0.115, 0.031) | 0.04 | 0.26 | |  | 0.002 (-0.004, 0.007) | | | 0.003 | 0.56 |
| Depressive symptoms | | -0.015 (-0.086, 0.057) | 0.04 | 0.69 | |  | 0.002 (-0.004, 0.007) | | | 0.003 | 0.57 |
| Wellbeing | | -0.068 (-0.141, 0.004) | 0.04 | 0.06 | |  | 0.004 (-0.002, 0.009) | | | 0.003 | 0.17 |
| Schizophrenia | | -0.052 (-0.125, 0.022) | 0.04 | 0.17 | |  | 0.004 (-0.001, 0.009) | | | 0.003 | 0.12 |
| Extreme BMI | | -0.048 (-0.118, -0.023) | 0.04 | 0.18 | |  | 0.003 (-0.002, 0.008) | | | 0.003 | 0.24 |
| Note: Analyses based on growth-curve mixed effects models, controlling for interactions between age and the polygenic score, interactions between age and sex, as well as the 10 principal components.  ^a^ Based on self-reported victimisation at 7, 10, and 12 years.  ^b^ Based on self-reported victimisation at 13, 15, and 17 years. | | | | | | | | | | | |

Self age 7 years

Peers age 7 years

Teacher 7 years

Self age 10 years

Peers age 10 years

Teacher 10 years

.65*

* *p* < .05. Estimates are standardized.

.37*

.60*

.58*

.43*

.72*

.63*

***Figure S1:*** *Two-factor confirmatory analysis (CFA)*
